# Exercise Prescription in Cardio-Oncology

**DOI:** 10.3390/jcm14113724

**Published:** 2025-05-26

**Authors:** Nicola Campana, Luca Fazzini, Clelia Donisi, Alessandro Nava, Michele Migliari, Martino Deidda, Andrea Pretta, Mario Scartozzi, Christian Cadeddu Dessalvi

**Affiliations:** 1 Department of Medical Sciences and Public Health, University of Cagliari, 09124 Cagliari, Italy; nicolacampana93@gmail.com (N.C.); luca.fazzini10@gmail.com (L.F.); cleliadonisi@gmail.com (C.D.); michele.migliari1@gmail.com (M.M.); martino.deidda@tiscali.it (M.D.); an.pretta@gmail.com (A.P.); marioscartozzi@gmail.com (M.S.); 2 Unità Clinico Operativa di Clinica Medica, University of Trieste, Piazzale Europa, 1, 34127 Trieste, Italy; nava.alessandro.14@gmail.com

**Keywords:** exercise prescription in oncology, cardiotoxicities, cardiorespiratory fitness, cardiovascular complications in cancer survivors

## Abstract

Numerous studies underscore the benefits of exercise prescription in both cardiology and oncology. Recently, emerging eviAlessandro Navadence has highlighted the value of exercise in cardio-oncology, demonstrating its protective effects against the decline in functional capacity and cardiovascular complications that may arise in oncology patients, either as a result of the disease itself or as a side effect of chemotherapy. The purpose of this review is to elucidate the protective mechanisms and cardiovascular clinical benefits conferred by exercise prescription in cancer patients. Additionally, it aims to delineate the principal current exercise protocols that have been validated or proposed, outlining their respective advantages and limitations. Finally, we will explore future perspectives, particularly the development of precision medicine, supported by advancements in AI, to facilitate the creation of novel, personalized exercise protocols tailored to specific patient populations.

## 1. Introduction

With the increasing survival rates among cancer patients, the growing burden of cardiovascular disease as a major cause of morbidity and mortality in this population has become increasingly evident. Cardio-oncology is a relatively new discipline, developed in response to the need to prevent, monitor, and manage cardiovascular complications related to cancer itself and to oncologic therapies, such as chemotherapy and radiotherapy. In parallel, the interest in physical exercise as a preventive and therapeutic tool has expanded significantly—first within cardiology and oncology independently and more recently within the field of cardio-oncology.

A growing body of evidence highlights how physical activity can help preserve functional capacity, improve quality of life, and reduce cardiovascular risks in cancer patients, both during and after treatment. However, the development and implementation of structured, individualized exercise protocols remains an ongoing challenge.

This review aims to provide a scientific and clinical rationale for the integration of physical exercise into cardio-oncology care pathways in light of current evidence and emerging opportunities in the field of precision medicine.

## 2. Exercise Prescription in Cardiology

Only a few cardiac conditions, primarily during the acute phase or shortly after hospital discharge, fail to derive symptomatic or prognostic benefits from structured exercise programs. Absolute contraindications include unstable angina, uncontrolled arrhythmias, symptomatic severe aortic stenosis or other significant valvular diseases, decompensated heart failure, and acute myocarditis or pericarditis. Relative contraindications typically require a temporary suspension of exercise until appropriate management is achieved. These include uncontrolled tachyarrhythmias or bradyarrhythmias, severe hypertension, and certain myocardial diseases still under diagnostic evaluation.

Exercise prescription in cardiology mainly applies to patients with coronary artery disease, in both chronic and acute settings according to rehabilitation programs, and to patients affected by heart failure with both a reduced and preserved ejection fraction.

### 2.1. Patients Undergoing Percutaneous Coronary Intervention

The exercise prescription for patients with coronary artery disease undergoing percutaneous coronary interventions should be included in a secondary prevention program aiming to reduce the residual cardiovascular risk, ultimately decreasing morbidity and mortality [1,2]. The latest ESC acute coronary syndrome guidelines strongly recommend a medically supervised, comprehensive, multidisciplinary exercise-based rehabilitation program, which should start as soon as possible after the index event, accompanied by lifestyle and nutrition management [3]. Supporting these recommendations, recent evidence has demonstrated that exercise-based cardiac rehabilitation programs significantly reduce cardiovascular mortality and hospitalizations, while enhancing the quality of life in patients with a history of myocardial infarction, coronary artery bypass grafting, or percutaneous coronary intervention [4]. However, despite guideline recommendations and well-established benefits, only a small proportion of patients with a clear indication for cardiac rehabilitation are actually enrolled in exercise-based programs [2].

### 2.2. Stable Coronary Artery Disease

Stable coronary artery disease refers to a chronic condition characterized by atherosclerotic plaques in the coronary arteries, which may limit myocardial blood flow without acute ischemia or clinical instability [5]. Unlike patients recovering from myocardial infarction, for whom structured exercise-based cardiac rehabilitation is strongly recommended, individuals with stable coronary artery disease receive less formalized exercise prescriptions from physicians [6]. Despite robust evidence supporting the beneficial effects of exercise training in chronic ischemic heart disease, including improvements in endothelial function, the myocardial oxygen supply–demand balance, and long-term cardiovascular outcomes [7,8], the routine prescription of supervised exercise programs in this setting is often deprioritized. This is primarily due to cost-effectiveness considerations and logistical constraints, with healthcare systems prioritizing post-myocardial infarction patients. Nevertheless, regardless of medical prescriptions or access to structured rehabilitation programs, all patients with stable coronary artery disease should be encouraged in regular physical activity tailored to their individual capabilities, as persistency and sustained exercise participation remain a fundamental component of cardiovascular risk reduction [9]. Indeed, stable coronary artery disease and heart failure often coexist; the exercise prescription in heart failure patients is discussed below.

### 2.3. Heart Failure

Exercise training is consistently shown to enhance exercise tolerance and the quality of life in patients with heart failure. Specifically, in individuals with heart failure with reduced ejection fraction (HFrEF), structured rehabilitation has been shown to improve both their exercise capacity and quality of life [10,11]. Additionally, meta-analyses exploring the prognostic impact of exercise training, reported an HF-related and all-cause hospitalizations reduction, particularly among patients who adhere closely to the prescribed program [12,13]. Current guidelines recommend exercise for all patients, suggesting supervised, exercise-based cardiac rehabilitation in patients with advanced disease, frailty, and multiple comorbidities [14].

Similarly, patients with HF with preserved EF (HFpEF), often characterized by stiff hearts, increased ventricular filling pressures, and exercise intolerance, may particularly benefit from regular and supervised physical activity, which leads to increases in cardiac output and peak oxygen consumption while also contributing to improving prognoses [15,16,17]. Conversely, a sedentary lifestyle promotes a decline in cardiac output and an impaired ability to enhance cardiac performance during exercise, ultimately leading to low-threshold dyspnea [17].

## 3. Exercise Prescription in Oncology

Cancer poses a significant global burden, both in terms of economic costs and human impact. Consequently, prevention remains the most desirable strategy, with physical activity representing a key modifiable factor in reducing cancer risk. Compelling evidence indicates that regular physical activity is associated with approximately a 20% reduction in the breast cancer incidence and a 40–50% reduction in the risk of colorectal cancer [18]. This highlights physical activity as a potential intervention for lowering cancer risk [19,20].

Substantial evidence supports the role of physical activity in improving survival outcomes among individuals with cancer. A 2017 systematic review by Cormie et al., encompassing 100 studies—including randomized controlled trials, meta-analyses, and prospective cohorts, demonstrated a consistent association between higher levels of post-diagnosis physical activity and a reduced cancer-specific mortality, recurrence, and all-cause mortality [21]. While many of the included RCTs were not specifically powered to assess survival endpoints, the overall evidence highlights a meaningful trend across diverse cancer populations. Supporting this, Beasley et al. conducted a pooled analysis of four prospective cohorts—including over 13,000 women with breast cancer—showing that an adherence to physical activity guidelines (≥10 MET-hours/week) was associated with a 27% reduction in all-cause mortality and a 25% reduction in breast cancer-specific mortality, although no significant effect on recurrence was observed [22]. In pediatric cancer survivors, similar findings were reported by Scott et al., who analyzed data from more than 15,000 adults treated for childhood cancer, revealing a 40% reduction in all-cause mortality among those who engaged in or increased vigorous physical activity over time [23]. In this context, the systematic review and meta-analysis conducted by Morishita et al. provides additional evidence, showing that exercise significantly reduces cancer-related mortality and recurrence, notwithstanding some limitations in the study design and heterogeneity [24]. Lifestyle modifications can positively influence survival patients’ outcomes, regardless of the type or stage of their illness. This idea is supported by a meta-analysis conducted by Rabbani and colleagues, which reviewed 98 cohort and case–control studies from around the world up to 30 November 2024 [25]. These studies involved a total of 1,461,834 patients and demonstrated positive results, with a pooled log hazard ratio of −0.31 (95% confidence interval: [−0.38, −0.25]; *p* < 0.001, I^2^ = 0%) [25]. The most common cancer types included breast, colorectal, lung, and prostate cancers. The analysis found that regular physical activity helps reduce body fat, enhances immune function, and regulates hormone levels, all of which contribute to lower rates of cancer recurrence and improved outcomes [26,27,28,29,30,31,32,33,34,35,36,37,38,39,40,41,42,43,44,45,46]. In particular, seven studies showcased the beneficial impact of regular exercise on survival rates in patients with breast, ovarian, colorectal, and other types of carcinoma [27,31,32,36,41,45,47]. However, six studies examined the relationship between exercise intensity and survival outcomes, and their findings were inconsistent, possibly due to the varying types of cancer patients enrolled [31,33,34,39,42,43]. Furthermore, various studies have emphasized the importance of the timing of physical activity in relation to the diagnosis of cancer [31,37,38,41,45].

Filis and colleagues conducted an umbrella review, analyzing 740 meta-analyses to explore the connection between physical activity and outcomes for cancer survivors. Notably, 90% of these meta-analyses included randomized controlled trials (RCTs), while the remaining 10% focused on cohort studies [48]. In the post-diagnosis phase for breast cancer patients, physical activity has been shown to reduce recurrence rates, mortality, fatigue, and depression, while also enhancing mental health, body strength, aerobic capacity, and promoting weight loss [49,50,51,52,53,54,55,56,57]. For prostate cancer patients, regular physical activity improves their cardiovascular fitness and quality of life, while reducing fatigue [58,59,60]. In the case of colorectal cancer, increased physical activity has been linked to lower mortality rates [61]. Furthermore, for lung cancer patients, pre-operative breathing exercises and physical activity have been shown to decrease hospital stays [62,63].

However, despite these consistent findings, there is a notable lack of data concerning other cancer types, including brain, head and neck, skin, gastric, pancreatic, liver, and gynecological cancers [48]. Therefore, further research is essential to better understand how physical activity influences pathological response rates, to identify the most effective type, dose, and intensity of exercise, and to define the specific characteristics of the patient populations that may benefit the most.

Additionally, it is well known that depression and an impaired quality of life (QoL) affect many cancer patients. Research has indicated that physical activities, particularly intense ones, can increase the release of β-endorphins, which may lead to reduced levels of depression and anxiety [64].

Over the years, several studies have investigated the effects of physical and nutritional interventions in patients with various types and stages of cancer, including breast, lung, colorectal, prostate, gastric, and liver cancer, as well as in cancer survivors [65,66,67,68]. A systematic review and meta-analysis conducted by Petros and colleagues examined these studies [69]. A total of 38 publications were included in the systematic review, highlighting an association between physical activity combined with nutritional interventions and improvements in mental health, along with reductions in the body mass index (BMI), insulin levels, HOMA-IR, C-reactive protein, and triglycerides. Additionally, there were enhancements in high-density lipoprotein (HDL) levels and improvements in the QoL, all of which have the potential to reduce cancer risks and increase survival rates [70,71]. A diet rich in protein, combined with resistance exercises, may help prevent the loss of lean muscle mass [72,73,74,75]. One notable limitation of this systematic review is the substantial heterogeneity in the physical and nutritional interventions administered across the various study populations [69].

Studies reporting positive outcomes have been published more frequently than those with null or negative results, potentially introducing publication bias. Nevertheless, the available evidence strongly supports the notion that physical activity provides a survival benefit in cancer populations, emphasizing its role as a key component of comprehensive oncologic care—even beyond cardiovascular outcomes. These findings highlight the need for clinical guidelines that promote the integration of physical activity interventions into cancer treatment to enhance survivorship.

## 4. Exercise in Cardio-Oncology

Thanks to advances in oncologic therapies, the number of cancer survivors continues to grow steadily each year. However, this increase in survival is accompanied by a rising burden of cardiovascular morbidity and mortality, largely due to the complex interplay between shared risk factors for cancer and cardiovascular disease, the direct effects of malignancy, and the cardiotoxicity induced by anticancer treatments, including both chemotherapy and radiotherapy. In response to this emerging clinical challenge, recent years have seen a growing number of studies focused on identifying strategies to reduce cardiovascular complications in cancer survivors. Among these strategies, growing attention has been directed toward assessing the role of exercise training in preserving cardiovascular health in cancer patients. An expanding body of evidence supports the effectiveness of structured exercise programs in mitigating the adverse effects related to cancer and its therapies, safeguarding both cardiac and vascular function, and ultimately contributing to improved clinical outcomes during and after oncologic treatment.

### 4.1. Main Mechanisms of Cardiovascular Protection Induced by Exercise in Cancer Patients

The mechanisms by which physical exercise prevents the development of cancer- or chemotherapy-related cardiovascular side effects are diverse and multifaceted.

#### 4.1.1. Positive Cardiac Remodeling

Positive cardiac remodeling results from a combination of adaptive mechanisms, including the development of physiological hypertrophy, which enhances the resistance to chamber dilation, as well as improvements in cardiomyocyte energetic efficiency and favorable modifications in myocardial vascularization.

Exercise-induced physiological hypertrophy is proportional, adaptive, and reversible, distinguishing it from pathological hypertrophy, which is marked by fibrosis, impaired contractile function, and the reactivation of the fetal gene program [76]. Exercise promotes physiological hypertrophy through multiple mechanisms. In response to physical training, cardiomyocytes undergo the enlargement of individual muscle cells, accompanied by an increase in the mitochondrial energy capacity. Furthermore, exercise has been shown to induce a tissue-level proliferation of already differentiated cardiomyocytes by inhibiting the transcription factor C/EBPβ and enhancing the expression of the CBP/p300-interacting protein with ED-rich carboxy-terminal domain-4 (CITED4) [77]. Exercise is also capable of activating cardiac progenitor cells that express C-kit and Sca1. These progenitor cells possess differentiation capabilities and self-renewal potential, thereby contributing to myocardial regeneration [78].

Among the main molecular pathways involved in the development of physiological hypertrophy, the PI3K/Akt/mTOR pathway plays a central role. This pathway is activated by extracellular growth factors, such as IGF-1, as well as by intracellular microRNAs. Its effects are further enhanced by the neuregulin-1/ErbB2/ErbB4 signaling cascade, which is also stimulated by physical exercise [79]. Another important mechanism implicated in the genesis of physiological hypertrophy involves nitric oxide (NO), which exerts cardioprotective effects, particularly in the setting of ischemic injury. In a murine model, numerous microRNAs have been identified as key regulators of cellular differentiation and proliferation by modulating gene expression post-transcriptionally through binding to specific mRNA targets. Among these, the miR-17-92 cluster has emerged as one of the most prominent contributors to these exercise-induced molecular processes [80,81].

Additionally, exercise enhances the calcium release and reuptake at the level of the sarcoplasmic reticulum, thereby preventing intracellular calcium overload [82].

It also improves the vascular profile by stimulating PGC-1α expression, promoting VEGF expression, and increasing the production of NO and interleukin-6 (IL-6), which collectively confer protection against cancer- and chemotherapy-induced ischemia [83].

#### 4.1.2. Modulation of Inflammation and Oxidative Stress

Physical activity plays a crucial role in reducing systemic inflammation and oxidative stress, both of which are exacerbated by cancer therapies. It enhances endogenous myocardial antioxidant defenses, thereby limiting the accumulation of chemotherapy-induced reactive oxygen species (ROS). Additionally, exercise promotes improved mitochondrial function and bioenergetic efficiency, while downregulating pro-apoptotic signaling pathways, ultimately contributing to cardiomyocyte survival and an improved cardiac resilience during oncologic treatments [84].

#### 4.1.3. Mitigation of Autonomic Dysfunction

Autonomic dysfunction is commonly observed in cancer patients, particularly in advanced stages of the disease, and it is associated with increased mortality, reduced exercise capacity, and heightened fatigue. Oncologic treatments, such as chemotherapy and radiotherapy, contribute to the development of autonomic dysfunction through cardiotoxic and neurotoxic effects, resulting in reduced parasympathetic tone and heightened sympathetic activity [85,86]. Exercise improves autonomic function by enhancing parasympathetic (vagal) tone and reducing sympathetic activity. This rebalancing contributes to better cardiovascular control, helping to manage therapy-induced autonomic dysfunction and its associated complications, such as arrhythmias, fatigue, and reduced exercise capacity [87,88].

### 4.2. Evidence Supporting Exercise in Cardio-Oncology

Over the past two decades, physical exercise has emerged as a key strategy in cardio-oncology to counteract cardiovascular complications related to cancer therapies. Structured exercise programs, particularly those combining aerobic and resistance training, have been shown to reduce the risk of major cardiovascular events and improve cardiorespiratory fitness—a strong predictor of mortality in cancer patients. Exercise also exerts beneficial effects on vascular function, blood pressure, lipid metabolism, and oxidative stress. Importantly, it may help protect against the direct myocardial injury induced by cardiotoxic agents like anthracyclines, contributing to the preservation of cardiac function and improved clinical outcomes.

#### 4.2.1. Cardiovascular Outcomes

An expanding body of evidence strongly supports the association between physical activity and a substantial reduction in cardiovascular events and mortality among cancer survivors. These benefits have been consistently observed in both observational studies and randomized controlled trials, underscoring the importance of incorporating physical activity into cardio-oncology care pathways to mitigate the long-term cardiovascular risk. The main studies are reported in Table 1.

In a prospective cohort study involving nearly 3000 women with nonmetastatic breast cancer, Jones et al. demonstrated that performing at least 9 MET-hours of physical activity per week was associated with a 23% reduction in overall cardiovascular events. Specifically, the incidence of coronary artery disease was reduced by 26%, while the occurrence of heart failure decreased by 29%. Importantly, a clear dose–response relationship was observed, with higher volumes of physical activity correlating with progressively lower rates of both ischemic and non-ischemic cardiovascular complications [89].

In a larger population-based cohort of over 39,000 five-year breast cancer survivors, Kim et al. found that engaging in ≥1000 MET-minutes per week of physical activity was associated with a 27% lower risk of cardiovascular disease. Notable reductions were observed for both coronary heart disease and stroke, with the most substantial benefits reported among individuals engaging in moderate-to-vigorous activity three to four times per week [90]. Similarly, Jung et al., in a nationwide study of nearly 270,000 cancer survivors, showed that both maintaining and initiating physical activity after a cancer diagnosis were significantly associated with reduced cardiovascular risk. Survivors who remained physically active experienced a 20% lower risk of myocardial infarction and a 16% lower risk of heart failure. Notably, individuals who began exercising only after diagnosis still achieved meaningful risk reductions, highlighting the reversibility of risk through behavioral intervention [91].

Taken together, these findings underscore the pivotal role of physical activity as an evidence-based strategy for reducing cardiovascular risk in cancer survivors. Exercise should be regarded not merely as a supportive measure to enhance physical and psychological well-being, but as a fundamental intervention with the potential to positively influence long-term clinical outcomes.

**Table 1:** A summary of the main studies that explored the impact of exercise on cardiovascular outcomes (↑ increase; ↓ decrease).

Year of Publication, Author, and Type of Study	Sample Size (*n*)	Patient Population Characteristics	Outcomes Observed in the Exercise Group (EG) vs. the Control Group (CG)
2024 Jung et al. (Observational cohort study) [91]	269,943	Different cancer survivor cohorts	↓ myocardial infarction ↓ heart failure
2020 Kim et al. (Observational cohort study) [90]	39,775	Breast cancer survivor	↓ cardiovascular disease ↓ stroke ↓ coronary artery disease
2017 Jones et al. (Observational cohort study) [89]	2973	Non-metastatic breast cancer survivors	↓ cardiovascular mortality ↓ coronary artery disease ↓ heart failure

#### 4.2.2. Cardiorespiratory Fitness (CRF)

Patients with cancer often exhibit reduced cardiorespiratory fitness (CRF), commonly assessed by measuring the peak oxygen uptake (VO_2_peak) during maximal exercise testing. Notably, cancer survivors typically display CRF levels that are 20–30% lower than those of healthy individuals of the same age, highlighting a substantial functional impairment that may persist well beyond the completion of treatment [92]. A recent meta-analysis conducted by Johansen’s research group—which included 44 studies encompassing a total of 1372 patients—demonstrated that systemic anticancer treatment is associated with a significant reduction in the VO_2_peak (weighted mean difference: −2.13 mL/kg/min). An even greater decline was observed two years after the completion of chemotherapy (−6.39 mL/kg/min). No significant differences were found between patient subgroups, including those with esophagogastric, breast, and colorectal cancers [93].

The mechanisms underlying the reduction in CRF are well established and clinically significant, arising from a complex interplay between central cardiovascular impairments—such as reduced cardiac output or vascular dysfunction—and peripheral alterations, including skeletal muscle atrophy, mitochondrial dysfunction, and impaired oxygen utilization. Importantly, these limitations in CRF may result not only from the direct physiological effects of cancer itself, but also from the toxicities induced by systemic anticancer therapies, particularly chemotherapy [94,95].

According to the Fick principle, a reduction in peak oxygen uptake (VO_2_peak) can be attributed to a decrease in either cardiac output or peripheral oxygen extraction.

Several studies have demonstrated that both patients who had not yet undergone chemotherapy and those who had completed treatment exhibited an impaired cardiac function at peak exercise, which contributed to the observed reduction in cardiorespiratory fitness (CRF) among cancer survivors. The reported decline in VO_2_peak—ranging from 5% to 33% compared to healthy controls—was clearly associated with a reduction in peak exercise cardiac output, estimated between 7% and 17% [95,96].

However, cardiac limitations should not be viewed as the sole contributors to the reduced exercise tolerance in cancer survivors. In a recent publication, Dillon et al. present an integrated model to explain the decline in cardiorespiratory fitness (CRF), emphasizing that both conventional cancer therapies (e.g., anthracyclines and radiotherapy) and emerging treatments (e.g., tyrosine kinase inhibitors, immunotherapy, and hematopoietic cell transplantation) can lead to peripheral toxicities that significantly contribute to the reduction in VO_2_peak, independently of left ventricular systolic dysfunction. The authors describe impairments across multiple components of the oxygen transport cascade, including the gas exchange, vascular vasodilatory capacity, skeletal muscle capillarization, mitochondrial function, and muscle composition (e.g., atrophy and myosteatosis), all of which collectively compromise systemic oxygen delivery and utilization [94]. The conceptual model proposed in 2024 was subsequently supported by findings from a meta-analysis published the following year by Johansen et al., which reinforced the role of peripheral mechanisms in exercise intolerance among cancer survivors. The analysis revealed that the observed reductions in VO_2_peak were significantly associated with a lower arteriovenous oxygen difference (a-v O_2_ diff), while no consistent relationship was found with the peak cardiac output. These findings suggest that the diminished exercise capacity in this population may be largely attributed to peripheral impairments in oxygen extraction and diffusion, rather than being solely driven by central cardiovascular limitations [93,97].

Understanding the underlying mechanisms and being able to identify reductions in CRF is crucial due to its prognostic significance. Extensive evidence has shown that reduced cardiorespiratory fitness (CRF), reflected by a diminished functional capacity, is a strong indicator of increased mortality, a lower quality of life, and a greater symptom burden in patients with cancer [98,99,100,101,102]. Evidence from a cohort of 1631 cancer patients who underwent cardiopulmonary exercise testing (CPET) post-diagnosis, with a follow-up exceeding four years, revealed that higher levels of functional capacity are significantly associated with lower mortality rates. Specifically, each incremental increase of 1 MET in exercise capacity corresponded to a 25% reduction in overall and cancer-related mortality and a 14% decrease in cardiovascular mortality [102]. Expanding upon these findings, the recent meta-analysis by Bettariga et al. (2025) synthesized data from 42 studies encompassing 46,694 cancer patients to investigate the relationship between CRF and mortality outcomes [103]. CRF was assessed using either maximal (CPET) or submaximal (6MWT) testing protocols. The analysis confirmed that higher CRF levels were significantly associated with improved survival, with each unit increase in CRF corresponding to an 18% reduction in cancer-related mortality. This protective association was especially prominent in patients with advanced-stage disease and in those with lung or gastrointestinal cancers. Together, these data underscore the critical role of CRF as both a prognostic marker and a potential target for therapeutic intervention in oncology.

Over the past two decades, a vast number of studies have investigated the effects of exercise on cardiorespiratory fitness (CRF) in individuals with cancer. Given the breadth of the available evidence, this section highlights the most relevant and widely cited meta-analyses that have shaped the current understanding of how exercise improves CRF across different oncologic populations, the main studies are reported in Table 2.

In 2011, Jones et al. conducted a meta-analysis including six randomized controlled trials (RCTs) with 571 adult patients diagnosed with breast, prostate, or colorectal cancer or lymphoma [104]. Supervised aerobic training led to a significant increase in the VO_2_peak (+2.90 mL·kg^−1^·min^−1^; 95% CI: 1.16–4.64), while a reduction was observed in the control group. The most notable improvements occurred in post-treatment programs shorter than 4 months, with a low incidence of adverse events (2.3%). Building on this early evidence, Zhou et al. (2016) analyzed nine studies involving 314 patients with acute leukemia [105]. Their findings confirmed that exercise significantly improved CRF (SMD = 0.45; 95% CI: 0.09–0.80; *p* = 0.01), especially when measured using the 12 min walk test.

In 2018, the field saw a further consolidation of evidence through several large-scale analyses. Scott et al. synthesized data from 48 RCTs, including 3632 patients with solid and hematologic tumors. They found that exercise increased the VO_2_peak by 2.80 mL·kg^−1^·min^−1^ compared to 0.02 in controls (WMD = 2.13; 95% CI: 1.58–2.67; *p* < 0.001), with consistent effects across different intervention types and timings [106]. That same year, Sweegers et al. performed an individual patient data meta-analysis on 3515 participants from 28 RCTs, confirming the positive impact of exercise on the VO_2_peak (+1.80 mL·kg^−1^·min^−1^; β = 0.28), especially among younger individuals, in supervised programs, and with a training frequency of ≥3 times/week [107]. In 2020, Maginador et al. focused on 493 women undergoing chemotherapy for breast cancer. They found that aerobic exercise resulted in a 9.97% increase in the VO_2_peak, in contrast to a 10.18% decline in the control group (d = 1.19; 95% CI: 0.45–1.94). Importantly, the benefits were observed only with vigorous-intensity protocols (64–90% VO_2_ peak; d = 1.47; *p* = 0.0009), with both continuous and interval modalities proving effective [108]. The evidence was further strengthened in 2021 by the meta-analysis of Pérez et al., which examined 25 studies encompassing 2515 patients across 22 cancer types. High-intensity training led to a significant improvement in CRF (SMD = 0.44; 95% CI: 0.25–0.64; *p* < 0.00001), with optimal effects found in pre-treatment interventions lasting more than 8 weeks, using aerobic formats with ≥20 min of vigorous activity per session [109].

Taken together, these successive meta-analyses demonstrate a coherent and growing body of evidence that consistently supports the role of structured exercise—especially aerobic and high-intensity modalities—in enhancing CRF across a wide range of cancer populations and treatment contexts.

**Table 2:** A summary of the main studies that explored the impact of exercise on cardiorespiratory fitness (↑ increase; ↓ decrease).

Year of Publication, Author, and Type of Study	Sample Size (*n*)	Patient Population Characteristics	Outcomes Observed in the Exercise Group (EG) vs. the Control Group (CG)
2021 Pérez et al. (Meta-analysis) [109]	2.515	Different cohorts of cancer survivors (breast, lung, colorectal, prostate, and testicular cancers)	Difference in peak VO_2_ favoring the exercise group over the control group (SMD = 0.44)
2020 Maginador et al. (Meta-analysis) [108]	493	Breast cancer survivor undergoing chemotherapy	Compared to baseline, peak VO_2_ ↑ 9.97% in EG and ↓ 10.18% in the CG
2018 Sweegers et al. (Meta-analysis) [107]	3.515	Different cohorts of cancer survivors (breast, genitourinary, hematological, gastrointestinal, gynecological, and pulmonary)	Compared to baseline ↑ VO_2_peak 1.80 mL·kg^−1^·min^−1^ in EG
2018 Scott et al. (Meta-analysis) [106]	3.632	Different cohorts of cancer survivors (Solid and hematological malignancies, breast cancer)	Compared to baseline ↑ VO_2_peak: 2.80 mL·kg^−1^·min^−1^ in EG
2016 Zhou et al. [105]	314	Acute leukemia patient	Improvement in CRF (SMD = 0.45) in EG as indicated by better performance on the walking test
2011 Jones et al. (Meta-analysis) [104]	571	Different cohorts of cancer survivors (breast, prostate, colon, and lymphoma)	Compared to baseline ↑ VO_2_peak 2.90 mL·kg^−1^·min^−1^ in EG

#### 4.2.3. Cardio Protection by Physical Exercise During Chemotherapy

Numerous clinical studies have been conducted following preclinical evidence showing that aerobic exercise could mitigate chemotherapy-induced cardiotoxicity. However, results from human studies remain heterogeneous and sometimes conflicting. The main studies are reported in Table 3.

Some trials have demonstrated a protective effect of exercise on left ventricular function. The ONCORE study by Díaz-Balboa et al. showed that although the left ventricular ejection fraction (LVEF) decreased in both groups, the reduction was significantly attenuated in the CORe group (a cardio-oncology rehabilitation program based on supervised physical exercise) compared to the control group [110]. The between-group difference in the LVEF change was −1.5% (95% CI: −2.9 to −0.1; *p* = 0.006), indicating a cardioprotective effect of exercise against chemotherapy-induced functional decline. A similar result was observed in the study by Chung et al., in which a moderate-to-high intensity training program initiated concurrently with chemotherapy maintained the LVEF in the exercise group (72.0% at baseline, 72.2% at 3 months, 68.3% at 6 months, and 70.4% at 12 months), whereas a significant decline was noted in the control group as early as 3 months (70.7% at baseline, 64.2% at 3 months, 64.8% at 6 months, and 62.2% at 12 months), with a statistically significant group-by-time interaction [111]. In the REH-HER study by Hojan et al., conducted in patients receiving trastuzumab, the LVEF remained stable in the exercise group (from 65.7 ± 5.0% to 64.9 ± 5.8%; *p* = 0.143), whereas it declined significantly in the control group (from 63.9 ± 2.7% to 59.8 ± 4.0%; *p* = 0.009), with a significant between-group difference in favor of exercise [112]. The study by Ma et al. reported a significant increase in the LVEF in the exercise group, from 55 ± 3.5% to 60 ± 2.9% (*p* < 0.05), while the control group showed a significant reduction from 51 ± 5.6% to 47 ± 2.6% (*p* < 0.05) [113].

Although some studies did not find significant changes in the resting LVEF, the overall findings suggest that exercise may still offer cardio protection against chemotherapy-induced damage. For example, the BREXIT study did not detect significant between-group differences in the resting LVEF, but showed substantial improvements in the contractile reserve during stress in the exercise group, with increased the LVEF, cardiac output (CO), and right ventricular function at both 4 and 12 months compared to the baseline [114]. The study by Antunes et al. (2023) investigated the effects of a supervised exercise program during anthracycline-based chemotherapy in women with early-stage breast cancer [115]. Although the left ventricular ejection fraction decreased in both groups, the decline was numerically smaller in the exercise group, without reaching statistical significance. However, the intervention led to a significant improvement in cardiorespiratory fitness, confirming that exercise is a safe and well-tolerated strategy during oncological treatment. This indicates that exercise enhances cardiac performance under an increased physiological demand. Similarly, in the study by Hornsby et al., the LVEF remained unchanged in both groups, but a significant improvement in cardiorespiratory fitness was observed only in the exercise group, suggesting a functional benefit not captured by conventional echocardiographic parameters [116]. The study by Howden et al. reported a significant overall reduction in the LVEF without significant between-group differences; nevertheless, exercise markedly reduced the incidence of functional impairment (defined as VO_2_peak <18 mL/kg/min), which occurred in 50% of control patients versus only 7% in the exercise group (*p* = 0.01), highlighting a substantial clinical benefit [117]. In the TITAN study by Kirkham et al., the LVEF remained unchanged in both groups, but the intervention led to significant improvements in the lipid profile (total cholesterol *p* = 0.002; LDL *p* < 0.01), which is potentially relevant for long-term cardiovascular prevention [118]. These findings suggest that even in the absence of significant changes in traditional resting echocardiographic parameters, exercise may attenuate chemotherapy-induced cardiotoxic effects by preserving functional capacity, enhancing the cardiac reserve, and improving the overall patient well-being.

The discrepancies observed among studies may be attributed to several methodological and clinical factors. A key determinant of efficacy is early initiation: studies in which exercise was initiated concurrently with chemotherapy, such as those by Chung and Ma, reported more evident cardioprotective effects. The duration and intensity of the intervention also appear to influence outcomes. The meta-analysis by Antunes et al. showed that only studies including at least 36 exercise sessions yielded significant improvements in the LVEF, whereas the overall analysis did not reach statistical significance [119]. This aligns with findings from the ONCORE study, where a supervised program spanning the entire chemotherapy course significantly attenuated the LVEF decline in the intervention group. Another critical aspect relates to the sensitivity of the outcome measures used to assess cardiac function. While the LVEF is the most widely adopted parameter, its ability to detect early subclinical changes is limited. In contrast, global longitudinal strain (GLS) has emerged as a more sensitive indicator. The meta-analysis by Linhares et al. reported a favorable effect of exercise on the GLS, with a significant between-group mean difference of +0.43% (95% CI: 0.03–0.82; *p* = 0.03), while the effect on the LVEF was weaker and characterized by high heterogeneity [120]. Adherence to the intervention is another important determinant. In the study by Kirkham et al., a protective effect on GLS was observed only in patients who attended at least 75% of the training sessions. In conclusion, exercise during chemotherapy appears to be a safe intervention with potential benefits in preserving cardiac function, particularly when assessed through more sensitive measures such as the GLS or stress-induced contractile reserve. However, the methodological and clinical heterogeneity across studies limits the ability to draw definitive conclusions. Future research should focus on well-designed, standardized interventions, sensitive cardiac biomarkers, and appropriate patient stratification to better define the role of exercise in the prevention of chemotherapy-related cardiotoxicity.

**Table 3:** A summary of the main studies demonstrating the cardioprotective effect of exercise against chemotherapy-induced damage (↑ increase; ↓ decrease).

Year of Publication, Author, and Type of Study	Sample Size (*n*)	Patient Population Characteristics	Outcomes Observed in the Exercise Group (EG) vs. the Control Group (CG)
2024 Díaz-Balboa et al. (RCT) [110]	122	Breast cancer patients planned to receive anthracyclines and/or anti-HER2	After chemotherapy (CT), a reduction in resting LVEF was observed in both groups, with an attenuated decline in the exercise group (EG) compared to the control group (CG).
2023 Foulkes et al. (RCT) [114]	104	Breast cancer patients planned to receive anthracyclines	After CT the EG showed superior stress echocardiographic parameters, less functional decline at 4 months, and lower troponin elevation, with no differences in resting LVEF compared to the CG.
2023 Antunes et al. (RCT) [115]	93	Early-stage breast cancer patients planned to receive anthracyclines	After CT the EG, unlike the CG, showed an improvement in cardiorespiratory fitness (↑ VO_2_peak) from baseline. A non-significant attenuation of LVEF reduction was observed in the EG.
2023 Kirkham et al. (RCT) [118]	74	Breast cancer patients planned to receive anthracyclines and/or anti-HER2	EG showed a more favorable lipid profile compared to CG. No differences were observed in resting LVEF.
2020 Chung et al. (RCT) [111]	32	Breast cancer patients planned to receive anthracyclines	After CT, LVEF improved in EG and worsened in CG. EG showed better resting LVEF, diastolic function, and less cardiac hypertrophy at 3, 6 and 12 months post-chemotherapy, as well as higher VO_2_peak at 12 months.
2020 Hojan et al. (RCT) [112]	68	Breast cancer patients planned to receive anti-HER2	Following CT, exercise stabilized resting LVEF and 6MWT performance in EG, while CG experienced a decline in both parameters.

#### 4.2.4. Additional Cardiovascular Benefits

In addition to improving cardiorespiratory fitness and the quality of life and mitigating cardiotoxicity, structured exercise interventions have been shown to exert broad and clinically meaningful effects on the cardiovascular profile of cancer survivors. These benefits encompass improvements in lipid and glucose metabolism, blood pressure control, body compositions, vascular function, and systemic inflammation. Together, these adaptations contribute to reducing the cardiovascular burden often induced by oncologic treatments, reinforcing the role of exercise as a comprehensive strategy for cardiovascular prevention in cancer patients. The main studies are reported in Table 4.

With regard to lipid metabolism, several randomized trials have demonstrated favorable modulations following exercise. In a 16-week supervised aerobic and resistance training program, Dieli-Conwright et al. observed an increase in HDL cholesterol of +21.6 mg/dL in breast cancer survivors [121]. Similarly, Lee et al. (2019) reported an HDL increase of +24.4 mg/dL, accompanied by reductions in LDL and total cholesterol [122]. In the TITAN trial, a year-long cardiac rehabilitation-based intervention led to decreases in total cholesterol (−0.5 mmol/L) and LDL (−0.6 mmol/L), suggesting a protective effect on lipid homeostasis even during cardiotoxic chemotherapy [118].

Exercise also elicited significant reductions in blood pressure, another key determinant of cardiovascular risk. Dieli-Conwright et al. reported a reduction of −15.2 mmHg in systolic blood pressure and −13.7 mmHg in diastolic blood pressure following the 16-week intervention [121]. These results were mirrored by Lee et al., who documented a similar −13.7 mmHg decrease in systolic pressure. The latter study also showed a mean reduction of 9.5 points in the Framingham risk score, corresponding to an 11% drop in the estimated 10-year cardiovascular risk [122].

Regarding glucose regulation and insulin resistance, structured exercise significantly improved glycemic control and insulin sensitivity. In the Dieli-Conwright study, exercise led to significant reductions in fasting glucose and insulin levels, alongside improvements in insulin resistance as indicated by HOMA-IR. Additionally, favorable changes were observed in adipokines and inflammatory markers, including increased adiponectin and decreased leptin, interleukin-6 (IL-6), and free testosterone [122]. These findings are supported by Zhu et al. who conducted a meta-analysis of 33 RCTs involving 2659 breast cancer survivors and reported a mean reduction in insulin levels (−4.98), as well as favorable changes in IGF-II and IGFBP-1 [49].

Improvements in body composition have also been consistently documented. Dieli-Conwright et al. observed reductions in the BMI (−5.5 kg/m^2^), total and trunk fat mass, and body weight (−4 kg), with significant effects on sarcopenic obesity [121]. In prostate cancer survivors treated with ADT and radiotherapy, Galvão et al. demonstrated an increase in appendicular muscle mass of +0.4 kg and improvements in functional capacity, as evidenced by a 19 s reduction in the 400 m walk test (*p* = 0.029), with gains sustained over 12 months [123].

Concerning vascular function, multiple studies have highlighted exercise-induced improvements in endothelial and arterial parameters. Beaudry et al., in a meta-analysis, found a mean increase of +1.3% in the flow-mediated dilation (FMD), which is indicative of enhanced endothelial responsiveness and associated with decreased cardiovascular risk [124]. Jones et al. demonstrated a reduction in arterial stiffness as measured by the pulse wave velocity [125]. In testicular cancer survivors, Adams et al. reported that high-intensity interval training over 12 weeks led to reductions in arterial stiffness, carotid intima–media thickness, systemic inflammation, LDL cholesterol, Framingham risk scores, and vascular age [126].

**Table 4:** A summary of the main studies showing the exercise-induced benefits on the cardiovascular profile in patients with cancer (↑ increase; ↓ decrease).

Year of Publication, Author, and Type of Study	Sample Size (*n*)	Patient Population Characteristics	Outcomes Observed in the Exercise Group (EG) vs. the Control Group (CG)
2023 Kirkham et al. (RCT) [118]	74	Breast cancer patients planned to receive anthracyclines and/or anti-HER2	↓ total cholesterol ↑ LDL cholesterol
2020 Jones et al. (RCT) [125]	51	Breast cancer survivors	Reduction in arterial stiffness (↓ aortic pulse wave velocity), along with improvements in CRF (↑ VO_2_ peak) and muscle strength.
2019 Lee et al. (RCT) [122]	100	Early-stage breast cancer survivors	↓ CVD risk ↓ blood pressure ↑ HDL cholesterol ↓ LDL cholesterol ↓ diagnosis of diabetes
2018 Beaudry et al. (Meta-analysis) [124]	163	After chemotherapy in breast and prostate cancer	Improvement in vascular endothelial function
2018 Dieli-Conwright et al. (RCT) [121]	100	Breast cancer survivors	↓ blood pressure ↓ triglycerides ↑ HDL cholesterol ↓ BMI ↓ fasting blood glucose ↓ metabolic syndrome z-score
2017 Adams et al. (RCT) [126]	63	Testicular cancer survivors	↓ CVD risk ↓ rest heart rate ↓ blood pressure ↓ arterial thickness and arterial stiffness ↑ postexercise parasympathetic reactivation ↓ inflammation ↓ LDL cholesterol
2013 Galvao et al. (RCT) [123]	100	Long-term prostate cancer survivors	↑ HDL levels, along with additional benefits such as improved CRF (↑ 6MWT distance) and increased muscle strength.
2016 Zhu et al. (Meta-analysis) [49]	2659	Breast cancer survivors	↓ insulin and insulin-like growth factor-II, ↓ BMI additional benefits such as improved quality of life, social well-being, and reduced depression and anxiety (as assessed by validated questionnaires).

## 5. Main Exercise Protocols in Cardio-Oncology

A comprehensive understanding of cancer development, its risk factors, and responses to personalized treatment strategies is increasingly recognized as a cornerstone of clinical practice, alongside the need to evaluate the specific physiological adaptations elicited by different exercise protocols in each individual patient [83].

The exercise prescription for cancer survivors should be individually tailored, considering factors such as their age, pre-treatment aerobic capacity, physical fitness, prior exercise experience, existing comorbidities, potential exposure to cardiotoxic agents or ionizing radiation, treatment-related responses, and any short- or long-term side effects; for instance, assessing for peripheral neuropathies and musculoskeletal issues is recommended before initiating any program [127].

The presence of pre-existing contraindications related to the patient’s cancer history—such as hematologic disorders, acute infections, or neurological conditions—should be carefully assessed during the initial evaluation and prior to each training session [128]. The suitability for engaging in physical activity should be carefully assessed in individuals with known cardiac abnormalities—such as structural heart defects, cardiomyopathies, or channelopathies—or if any concerns arise during risk stratification or clinical evaluation in order to ensure safe participation and reduce the risk of adverse cardiac events. Due to the increased cardiovascular risk compared to the baseline population, a functional assessment for risk stratification at baseline could be recommended, including the global cardiovascular risk [127].

Focusing on the protocols available in the literature, there is still no strong evidence to establish specific protocols focused on cardio-oncology.

The American College of Sports Medicine has published a document, developed during a roundtable discussion, providing guidelines for exercise prescriptions in oncology patients. In this document, published in 2010 by Schmidt et al., the authors reviewed evidence addressing the safety and feasibility of exercise during and after cancer treatment. They also examined whether exercise influences the treatment efficacy, symptom burden, toxicities, treatment tolerance, long-term adverse effects, and outcomes such as recurrence and survival. Based on the reviewed studies, this document confirmed the safety and beneficial effects of physical activity in cancer patients, recommending a model that includes moderate-intensity exercise for 75 min per week or light-intensity exercise for 150 min per week. Additionally, it suggests combining aerobic activity with strength/resistance training 2–3 times per week, targeting the major muscle groups [129]. However, despite that exercise was shown to be associated with better outcomes, as discussed above, no strong evidence of difference is reported between different exercise protocols and intensities. Considering that, exercise prescriptions should be patient-tailored considering their cardiovascular comorbidities, functional status, frailty, and overall fitness status. Different protocols might be applied according to the cancer. The exercise tolerability may differ significantly between breast cancer patients receiving anthracyclines (usually young women) and older lung cancer patients undergoing immunotherapy. Accordingly, the type of cancer and the overall functional status play a pivotal role in prescribing the exercise protocol intensity and duration. Below are the main aerobic and resistance protocols which might be applied.

### 5.1. Aerobic Training

A preliminary evaluation is recommended before initiating a prescribed aerobic training program, both to further stratify the cardiovascular risk—including potential ischemic or arrhythmic abnormalities—and to gather functional parameters necessary for defining appropriate training intensity zones [130]. Special caution should be taken in cases of baseline pump dysfunction. In these patients, a slow start with lower training loads is recommended, with gradual increases based on the individual’s condition. Monitoring the level of cardiac compensation before each training session is crucial to ensure safety and appropriate progression. In order to prescribe and supervise aerobic exercise in accordance with the recommendations of major scientific societies, it is essential to determine the exercise intensity thresholds that correspond to moderate and vigorous levels for each individual patient. To this end, a cardiopulmonary exercise test (CPET)—the gold standard for functional evaluation—should be performed prior to initiating an aerobic training program. If unavailable, a conventional exercise stress test with continuous ECG monitoring via telemetry may be used as an alternative, as it still allows for the assessment of the arrhythmic burden and the detection of potential ST-segment abnormalities during exertion. A 6 min walking test (6MWT) is a suitable alternative to evaluate the submaximal capacity both in cardiac and cancer patients [131]. While executing the aforementioned tests, it is important to systematically assess the patient’s perceived fatigue using a structured approach. The most commonly used method is the Rating of Perceived Exertion (RPE) scale, with the Borg Scale. Defining intensity domains is a fundamental aspect of training planning, with four main levels identified: low, moderate, high, and very high.

Based on the current literature, moderate-intensity exercise is generally set at a level just above the first ventilatory threshold (VT1) or lactate threshold (LT), while high-intensity exercise is positioned just below the second ventilatory threshold (VT2). The corresponding values for the maximum heart rate and peak VO_2_ can be tailored to each individual.

Considering heart rate domains, various methods exist in the literature to determine HR thresholds corresponding to a predefined intensity. Training intensity can also be defined as a percentage of the maximum heart rate (HRmax), which can be determined through an exercise test or estimated using the formula HRmax = 220 − age. However, relying on predicted HRmax values is not recommended due to the high variability and standard deviation in the relationship between age and the HR max. As an alternative, exercise intensity can be expressed as a percentage of the heart rate reserve (HRR), which accounts for the difference between the peak HR and resting heart rate (HR rest). This method, known as the Karvonen formula, calculates training intensity by applying a percentage of the HRR and adding it to the HR rest, providing a more individualized and accurate measure of exercise effort (especially in patients on medication that influences their normal heart rate, such as beta blockers or antiarrhythmic drugs) [132].

Aerobic activity can be structured using two main protocols: high-intensity interval training (HIIT) and moderate-intensity continuous training (MICT). Studies suggest both in cancer and cardiopathic patients, there is a tendency for HIIT to be more effective than MICT in improving the relative peak oxygen consumption (relVO_2_peak) [133]. 

The steps to follow for a methodological prescription of aerobic exercise in cancer patients are outlined in Table 5.

### 5.2. Resistance Training

As cancer treatments often lead to muscle wasting, fatigue, and decreased physical function, resistance training can counteract these effects, improving the overall quality of life [134]. Moreover, maintaining adequate muscle mass may enhance the efficacy of oncological treatments [135]. As highlighted in several position papers, the initial step in prescribing resistance training is to assess maximal strength, commonly defined as the one-repetition maximum (1 RM)—the highest amount of weight an individual can lift in a single repetition—which also reflects the maximal force that can be produced during a single voluntary muscle contraction. For more fragile patients or those new to training, less invasive methods for estimating maximal strength are available, such as the Brzycki formula [136]. Once the necessary value for determining the loading unit is obtained, the training volume, defined as the total amount of work performed (sets × repetitions × load), should be specified. Manipulating resistance training variables is essential to tailor the stimulus of each exercise. Beyond the load and repetitions, several factors can influence adaptations. These include training to failure or not, the range of motion (full or partial), the specific phase of the movement (e.g., muscle length), and load displacement. Other key elements are the time under tension, the duration and position of isometric phases, the type of contraction (concentric, eccentric, or both), internal vs. external focus, and inter-set rest. Adjusting one or more of these variables allows for increasing, maintaining, or reducing the training stimulus [137].

Based on the mode of execution, resistance training can be categorized into three main types: strength, hypertrophy, and endurance training [138].

A resistance training program combining muscular strength and muscular endurance components has been shown to produce musculoskeletal benefits, along with improvements in the cardiopulmonary function and health-related quality of life [139].

Hypertrophy-oriented resistance training not only enhances physical appearance and self-esteem but also supports metabolic health and functional independence. Moreover, this type of training may help counteract cancer-related cachexia by attenuating systemic inflammation and preserving lean body mass [140].

In a position stand by the American College of Sports Medicine (ACSM), it is recommended that novice to intermediate individuals perform resistance training with loads corresponding to 60–70% of their one-repetition maximum (1 RM) for 8–12 repetitions. For advanced individuals, cycling training loads between 80 and 100% of 1 RM is advised to maximize muscular strength [138].

To prevent adverse effects, it is recommended to avoid excessive fatigue and monitoring symptoms. Dyspnea, dizziness, excessive fatigue, or abnormal heart rates should prompt an immediate reassessment. Alongside this, in training programs it is important to include stretching and flexibility sessions, simultaneously working on muscle relaxation and proprioceptive development. Adequate recovery strategies stressing correct rest, hydration, and nutritional support should be planned. The periodization of the workload must be carefully planned for a correct progression of training.

The steps to follow for a methodological prescription of aerobic exercise in cancer patients are outlined in Table 6.

## 6. Future Perspectives

Numerous studies, as cited in this review, have demonstrated the beneficial role of physical exercise within the field of cardio-oncology. However, the current body of evidence is marked by substantial heterogeneity in the modalities of exercise prescription. In response to this, leading international societies and expert authors have proposed standardized models aimed at facilitating the clinical application of exercise interventions in cardio-oncological settings.

Future directions, however, envision a transition away from a universal prescription model toward the development of tools capable of phenotyping patients and tailoring exercise protocols accordingly. In this context, Scott et al. argue for a paradigm shift from standardized protocols to precision-based exercise prescriptions that account for the clinical, physiological, and tolerance variability observed among cancer patients [141]. Their framework emphasizes individualized assessments through cardiopulmonary testing and physiological profiling, supported by machine learning and predictive modeling, with the goal of optimizing the effectiveness, safety, and tolerability of interventions. Similarly, Sasso et al. propose a theoretical framework designed to move beyond generalized protocols, often used in clinical trials, toward personalized training regimens grounded in the physiological assessment of each patient [142]. Their approach underscores key training principles, such as individualization, specificity, progressive overload, and recovery, to enhance physiological adaptations and clinical outcomes while preserving adherence and safety.

A personalized exercise prescription requires the careful identification of intended outcomes and patient phenotyping, considering the cancer type, treatment history, cardiovascular comorbidities, mental health, and overall cardiometabolic risk. Artificial intelligence (AI) may serve as a key enabler in this process, supporting the tailoring of interventions to achieve predefined objectives and improve long-term outcomes.

RC Deo clearly highlights the dual utility of machine learning in medicine, both for predicting clinical outcomes (via supervised learning) and for discovering new phenotypes or patient subgroups (via unsupervised learning). Despite a growing number of studies, real-world clinical impact remains limited, largely due to a lack of large, standardized, and richly annotated datasets. In the context of increasingly individualized exercise-based cardiovascular rehabilitation, AI is emerging as a critical tool [143]. As noted by Halasz and Piepoli, combining wearable device data with clinical and laboratory parameters through machine learning could enable fully personalized training protocols. This approach may overcome the limitations of standardized prescriptions by improving efficacy, adherence, and safety [144]. For instance, the work by Chen et al. introduces an AI-driven system for exercise prescriptions targeting the “sub-healthy” population—individuals at risk but not yet diagnosed with overt disease. Using data from a fitness app with over 100,000 users, the system generated individualized exercise plans based on variables such as age, sex, body weight, and resting heart rate. Although efficacy was assessed indirectly through improvements in the resting HR, clinical endpoints were not included, and the model still awaits validation in real-world healthcare settings [145]. Gao et al. propose a clinically oriented protocol designed to develop an interpretable AI-based system for exercise prescription in cardio-oncology. The study will involve 600 patients with stage I cancer or cancer survivors, who will be followed over a four-year period. Personalized training programs will be tailored based on clinical parameters, lifestyle factors, and cardiovascular data. The AI system will utilize interpretable models—including decision trees, support vector machines (SVMs), and neural networks augmented with SHAP values—to provide transparent and explainable recommendations. The ultimate goal is to enhance the transparency, effectiveness, and clinical applicability of personalized exercise interventions in the cardio-oncology setting [146].

Future challenges in the field of cardio-oncology include several key areas that require further investigation and development. First, a major priority is the identification of the most relevant risk factors for accurate patient stratification. While clinical and functional parameters remain essential, the incorporation of biomarkers—alongside emerging omics and genomics technologies—is anticipated to play an increasingly important role in refining risk assessments and personalizing interventions.

Second, there is a significant gap in the evidence regarding the long-term safety of exercise interventions and the durability of their clinical benefits, particularly in cancer survivors who present with persistent treatment-related cardiotoxicity. Addressing this gap will require pragmatic randomized controlled trials specifically targeting cancer patients at an elevated risk for cardiovascular complications.

Third, as artificial intelligence continues to advance, it is imperative to determine which AI tools can be reliably and safely integrated into clinical practice. This necessitates the development and validation of high-quality, standardized datasets that can support robust and interpretable AI-driven decision-making.

Finally, the field must prioritize the design and execution of rigorous clinical trials aimed at validating specific exercise protocols across clearly defined patient subgroups. Such efforts are essential to move from generalized recommendations toward the implementation of precision exercise prescriptions as a standard component of cardio-oncology care.

## 7. Conclusions

The integration of structured exercise into cardio-oncology represents a critical opportunity to enhance both cardiovascular and oncologic outcomes in cancer patients. A robust body of evidence supports its role in improving cardiorespiratory fitness, attenuating treatment-related cardiotoxicity, and reducing cardiovascular and all-cause mortality. However, despite these established benefits, exercise prescription remains insufficiently personalized and underutilized in routine clinical care. Emerging models based on precision medicine, supported by artificial intelligence, offer the potential to tailor exercise interventions to individual patient profiles, maximizing therapeutic efficacy while ensuring safety. Moving forward, the development and validation of phenotype-driven protocols through randomized clinical trials will be essential. A multidisciplinary approach—uniting oncologists, cardiologists, exercise physiologists, and data scientists—will be key to implementing precision exercise as a standard component of comprehensive cardio-oncology care.

## Figures and Tables

**Table 5:** Multistep approach to aerobic exercise prescription in patients with cancer.

Step 1: define cardiovascular and cancer therapy-related cardiotoxicity risk		
Step 2: define training zones: %VO_2_ max, %HRR, %HRmax, RPE		
Step 3: prescribe exercise	Frequency and duration	- 150–300 min/week of moderate-intensity aerobic exercise or
		- 75–150 min/week of vigorous-intensity aerobic activity or
		- equivalent combination of both (30–60 min/session)
	Intensity	- Moderate: Borg Scale 12–13; 40–69% peak VO_2_; 55–74% peak HR; 40–69% HRR
		- Vigorous: Borg Scale 14–16; 70–85% peak VO_2_; 75–90% peak HR; 70–85% HRR

**Table 6:** Multistep approach to resistance training prescription in patients with cancer.

Define cardiovascular and cancer therapy-related cardiotoxicity risk		
Step 1: estimate maximal strength	Define Maximum Repetition (RM)	
	For more fragile patients less invasive methods for estimating maximal strength are available, such as the Brzycki formula	
Step 2: prescribe exercise	Load	Muscular strength: 60–70% 1 RM (novice/intermediate) and 80–100% 1 RM (advanced)
		Muscular Hypertrophy: 70–85% 1 RM (novice/intermediate) and 70–100% 1 RM (advanced)
		Muscular Endurance: <70% 1 RM
	Volume	Muscular strength: 1–3 sets of 8–12 reps (novice/intermediate) and 2–6 sets of 1–8 reps (advanced)—1–3 min recovery
		Muscular hypertrophy: 1–3 sets of 8–12 reps (novice/intermediate) and 3–6 sets of 1–12 reps (advanced)
		Muscular endurance: 2–4 sets of 10–25 reps
	Intensity	Muscular strength: 2–3 days/week (novice), 3–4 days/week (intermediate), and 4–6 days/week (advanced)
		Muscular hypertrophy: 3–4 days/week (split routine for intermediate/advanced)
		Muscular endurance: 2–3 days/week (full body)

## Abbreviations

The following abbreviations are used in this manuscript:

6MWT	European Society of Cardiology
BMI	6 min walking test
CPET	body mass index
CRF	cardiopulmonary exercise test
ESC	cardiorespiratory fitness
HDL	High-density lipoprotein
HFpEF	Heart Failure with preserved Ejection Fraction
HFrEF	heart failure with reduced ejection fraction
IGF-II	insulin like growth factor 2
IGFBP-1	Insulin-like Growth Factor Binding Protein 1
LDL	Low Density Lipoprotein
LVEF	left ventricular ejection fraction
MET	Metabolic Equivalent of Task
peak VO2	peak oxygen uptake
QoL	quality of life
RCTs	randomized control trials
